# Prognostic Impact of Ground-Glass Opacity in Clinical Stage IA Non-Small Cell Lung Cancer With Interstitial Lung Abnormalities

**DOI:** 10.1093/icvts/ivaf260

**Published:** 2025-10-31

**Authors:** Norifumi Tsubokawa, Takahiro Mimae, Takeshi Mimura, Atsushi Kagimoto, Atsushi Kamigaichi, Yoshihiro Miyata, Morihito Okada

**Affiliations:** Department of Surgical Oncology, Hiroshima University, Hiroshima 734-8551, Japan; Department of Surgical Oncology, Hiroshima University, Hiroshima 734-8551, Japan; Department of General Thoracic Surgery, National Hospital Organization Kure Medical Center and Chugoku Cancer Center, Kure 737-0023, Japan; Department of General Thoracic Surgery, National Hospital Organization Kure Medical Center and Chugoku Cancer Center, Kure 737-0023, Japan; Department of Surgical Oncology, Hiroshima University, Hiroshima 734-8551, Japan; Department of Surgical Oncology, Hiroshima University, Hiroshima 734-8551, Japan; Department of Surgical Oncology, Hiroshima University, Hiroshima 734-8551, Japan

**Keywords:** non-small cell lung cancer, ground-grass opacity, interstitial lung abnormalities

## Abstract

**Objectives:**

Ground-glass opacity (GGO) component is a favourable prognostic factor in non-small cell lung cancer (NSCLC), whereas NSCLC with interstitial lung abnormalities (ILA) generally has poorer prognoses. We investigated the clinical significance of GGO in patients with NSCLC and ILA.

**Methods:**

Among 1319 patients who underwent pulmonary resection for clinical stage IA NSCLC at 2 institutions between 2010 and 2020, we retrospectively assessed 216 patients with ILA based on preoperative CT. Patients were divided into 2 groups: pure solid tumours without GGO and subsolid tumours with GGO.

**Results:**

Among 216 patients with ILA, 146 (68%) had pure solid tumours and 70 (32%) had subsolid tumours. Subsolid tumours had significantly better prognoses than pure solid tumours (5-year overall survival, 69.7% vs 48.6%, *P* = .0008; 5-year recurrence-free survival, 69.7% vs 42.3%, *P* < .0001). Recurrence occurred in 4 patients (6%) with subsolid tumours and 41 (28%) with pure solid tumours. Although the 5-year cumulative incidence of lung cancer deaths was significantly lower in subsolid tumours than in those with pure solid tumours (2.6% vs 23.6%, *P* = .0011), an increase in other causes of mortality after 2 years post-surgery in subsolid tumours resulted in a comparable 5-year cumulative incidence of other causes of death (28.4% vs 36.1%, *P* = .260).

**Conclusions:**

In clinical stage IA NSCLC with ILA, subsolid tumours have a lower lung cancer mortality than pure solid tumours; however, higher other-cause mortality after 2 years contributes to poorer overall survival. Optimizing comorbidity management may improve long-term prognosis.

## Graphical abstract

**Figure:**
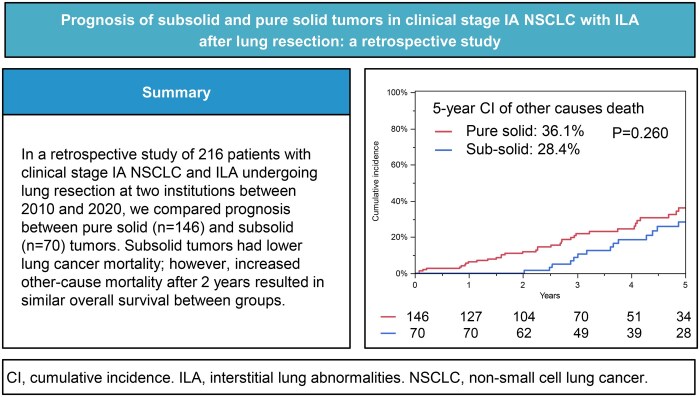

## INTRODUCTION

Interstitial lung abnormalities (ILA), representing a wide range of pulmonary fibrotic disorders, are reported to be associated with an increased risk of lung cancer.^1^^,^^2^ For early-stage non-small cell lung cancer (NSCLC) with ILA, surgical resection is often considered in carefully selected patients; however, the prognosis remains poor owing to multiple challenges. Owing to impaired pulmonary function and underlying fibrosis, patients cannot undergo anatomical resection with lymph node dissection^3^ and adjuvant therapies, including novel immunotherapy or tyrosine kinase inhibitors.^4–6^ Furthermore, postoperative acute exacerbation of ILA is highly fatal, worsening the prognosis.^2^^,^^7^^,^^8^

To improve the outcomes of patients with NSCLC and ILA, it is essential to understand the characteristics of ILA-associated lung cancer better. Lung cancer with ground-glass opacity (GGO) has a better prognosis than pure solid lung cancer.^9^^,^^10^ However, patients with ILA are known to have more aggressive tumours than those without ILA,^11^ and it remains unclear whether this also applies to lung cancer with GGO. Therefore, this study aimed to investigate the clinical significance of GGO in patients with NSCLC and ILA, focusing on its association with the prognosis.

## METHODS

This study was approved by the Institutional Review Boards of Hiroshima University (E2022-0125; August 25, 2022) and the National Hospital Organization Kure Medical Center and Chugoku Cancer Center (2022-28; September 1, 2022). Owing to the retrospective nature of the research, the requirement for informed consent was waived. Any collection and storage of data or biological material from research participants for multiple and indefinite use should be consistent with requirements outlined in the WMA Declaration of Taipei. A research ethics committee must approve the establishment and monitor ongoing use of such databases.

### Study cohort

Patients who underwent complete resection for clinical stage IA NSCLC at Hiroshima University and the National Hospital Organization Kure Medical Center and Chugoku Cancer Center between January 2010 and December 2020 were retrospectively reviewed. Those with preoperative high-resolution CT (HRCT) were assessed for the presence of ILA. Patients diagnosed with ILA on HRCT were included in this study. For supplementary analysis, we also compared survival and recurrence between patients with and without ILA, stratified by tumour type (pure solid vs subsolid). All patients underwent a preoperative evaluation that included HRCT and F-18-fluorodeoxyglucose positron emission tomography/CT. Each case was thoroughly reviewed by a multidisciplinary tumour board comprising experts in surgical and medical oncology, pulmonology, radiology, and pathology. Tumour staging was determined based on the TNM Classification of Lung and Pleural Tumours (8th edition).^12^

The surgical approach and procedures were selected based on the tumour and the clinical condition of the patient. For peripheral tumours, wedge resection or segmentectomy was performed in cases where complete tumour resection was feasible. Wedge resection was primarily indicated for GGO-dominant peripheral tumours or in patients with severely impaired pulmonary or cardiac function. If sublobar resection resulted in insufficient surgical margins, additional resection or conversion to segmentectomy or lobectomy was considered. Systematic lymph node dissection was performed in both segmentectomy and lobectomy, but not in wedge resection.

### Subsolid tumours and pure solid tumours

Tumours were classified based on preoperative HRCT. Ground-glass opacity was defined as an area of increased lung attenuation where the underlying vascular structures remained visible. The solid component was measured as the largest dimension of the dense region on lung window images, excluding any GGO regions. Tumours entirely composed of solid components were categorized as pure solid tumours, whereas those containing any GGO component were classified as subsolid tumours.

### Classification of ILA

Interstitial lung abnormalities were identified through preoperative HRCT. Based on the 2011 American Thoracic Society, European Respiratory Society, Japanese Respiratory Society, and Latin American Thoracic Association classifications,^13^ ILA patterns were classified into 3 groups: usual interstitial pneumonia (UIP) pattern, possible UIP pattern, or inconsistent with UIP pattern.

### Statistical analysis

Categorical variables were summarized as counts with percentages (*n*, %), while continuous variables were reported as medians with interquartile range. To compare baseline characteristics, the Mann-Whitney *U* test was applied for continuous variables, while Fisher’s exact test was used for categorical variables. Overall survival (OS) was defined as the duration from the surgery date to death from any cause or the last recorded follow-up. Recurrence-free survival (RFS) was determined as the duration from the surgery day to recurrence, death from any cause, or the last recorded follow-up. Locoregional recurrence was defined as tumour relapse within the ipsilateral thorax, including resection margins of the lung or bronchus, hilar and mediastinal lymph nodes, and malignant pleural effusions. Recurrence occurring outside these areas was considered distant recurrence. Survival outcomes were estimated using the Kaplan-Meier method and compared using the log-rank test. Regarding cox proportional hazards, covariates for multivariable analysis were selected based on their known clinical relevance and potential for confounding, including age, sex, smoking status, clinical T stage, surgical procedure, and pathological findings. For multivariable analysis, a backward stepwise method to select variables was employed. For time-to-event outcomes, a competing risk framework was implemented to estimate the cumulative incidence of recurrence or death, accounting for competing risks. Patients who were alive and recurrence-free at the final follow-up were censored, and the differences between groups were assessed using the Gray method. The proportional hazards assumption was visually evaluated using Kaplan-Meier and cumulative incidence curves. In addition, there were no missing values in the baseline covariates; thus, no imputation was required. All statistical analyses were performed using JMP (version 17.0; SAS Institute) and EZR version 1.51 (Saitama Medical Center, Jichi Medical University), a graphical user interface for R (R Foundation for Statistical Computing). Statistical significance was set at *P* < .05.

## RESULTS

### Patient characteristics between subsolid and pure solid tumours in patients with ILA

A total of 1319 patients who underwent complete resection for clinical stage IA NSCLC were included in this study. Among them, 1103 patients did not exhibit radiological findings of ILA (non-ILA). The remaining 216 patients were diagnosed with ILA based on preoperative HRCT (**Figure S1**).

Among the 216 patients with ILA, 146 (68%) had pure solid tumours and 70 (32%) had subsolid tumours. The clinical and pathological characteristics of pure solid and subsolid tumours in patients with ILA are shown in **Table 1**. Patients with subsolid tumours had significantly fewer males, fewer smokers, smaller solid tumour size, lower maximum standardized uptake value (SUVmax), lower clinical T factor, and higher predicted diffusing capacity for carbon monoxide than those with pure solid tumours (*P* = .0004, *P* < .0001 *P* < .0001, *P* < .0001, *P* < .0001, *P* = .036, respectively). Additionally, UIP pattern was more frequently observed in pure solid tumours than in subsolid tumours (*P* = .0004). Patients with pure solid tumours were more likely to undergo wedge resection than those with subsolid tumours (*P* = .0012). Regarding pathological factors, subsolid tumours had a higher proportion of lepidic-predominant adenocarcinoma and lower positive rate of pleural, lymphatic, and vascular invasion than pure solid tumours (*P* < .0001, *P* = .0014, *P* = .0029, *P* = .0001, respectively). The proportion of pathological lymph node metastases was comparable between the 2 groups (*P* = .495).

**Table 1.** Characteristics of Patients with ILA with Pure Solid Tumors and Subsolid Tumors

	Pure solid	Subsolid	*P*-value
	*n* = 146	*n* = 70	
Age, year, median (IQR)	74 (70-78)	74 (68-79)	.702
Sex (male), *n* (%)			.0004
Female	27 (18%)	29 (41%)	
Male	119 (82%)	41 (59%)	
Smoking history, yes, *n* (%)	139 (95%)	52 (74%)	<.0001
Solid tumour size, mm, median (IQR)	18.5 (15-22)	12.5 (7.0-20)	<.0001
SUV max, median (IQR)	4.9 (3.1-7.5)	1.8 (1.2-2.7)	<.0001
Consolidation to tumour ratio, *n* (%)			–
≤50%	–	25 (35%)	
>50%	–	45 (64%)	
Clinical T factor, *n* (%)			<.0001
cT1mi	0	7 (10%)	
cT1a	9 (6%)	18 (26%)	
cT1b	81 (55%)	29 (41%)	
cT1c	56 (38%)	16 (22%)	
Vital capacity, L, median (IQR)	2.94 (2.49-3.32)	2.87 (2.40-3.73)	.235
Forced expiratory volume in 1 s, L, median (IQR)	2.07 (1.77-3.43)	2.08 (1.80-2.35)	.774
Predicted diffusing capacity for carbon monoxide, %, median (IQR)	56.5 (42.8-75.3)	62.8 (50.6-83.2)	.036
ILA pattern on HRCT, *n* (%)			.0004
UIP	48 (33%)	7 (10%)	
Possible UIP	62 (42%)	36 (50%)	
Inconsistent with UIP	36 (25%)	29 (40%)	
Surgical procedures, *n* (%)			.0012
Wedge resection	60 (41%)	14 (20%)	
Segmentectomy	21 (14%)	22 (31%)	
Lobectomy	65 (45%)	34 (49%)	
Pathological T factor, *n* (%)			<.0001
pTis + T1mi	2 (1%)	17 (24%)	
pT1	98 (67%)	44 (63%)	
pT2	36 (25%)	6 (9%)	
pT3	8 (5%)	2 (3%)	
pT4	2 (1%)	1 (1%)	
Pathological N factor, *n* (%)			.495
pN0	119 (95%)	56 (92%)	
pN1	4 (3%)	3 (5%)	
pN2	1 (1%)	2 (3%)	
Histology, *n* (%)			<.0001
Adenocarcinoma	59 (40%)	65 (93%)	
Squamous cell carcinoma	75 (51%)	3 (4%)	
Others	4 (7%)	2 (3%)	<.0001
Adenocarcinoma predominant subtype			<.0001
Lepidic	4 (7%)	36 (55%)	
Papillary/acinar	42 (72%)	29 (45%)	
Micropapillary/solid	8 (14%)	0	
Others	4 (7%)	0	
Pleural invasion, *n* (%)	35 (24%)	5 (7%)	.0014
Lymphatic invasion, *n* (%)	33 (23%)	5 (7%)	.0029
Vascular invasion, *n* (%)	42 (29%)	5 (7%)	.0001
Postoperative complications, *n* (%)	34 (23%)	17 (24%)	.872
Postoperative respiratory complications, *n* (%)	27 (19%)	12 (17%)	.809

Abbreviations: HRCT, high-resolution CT; ILA, interstitial lung abnormality; IQR, interquartile range; SUVmax, maximum standard uptake value; UIP, unusual interstitial pneumonia.

### Prognosis between subsolid and solid tumours in patients with ILA

**Figure 1.**
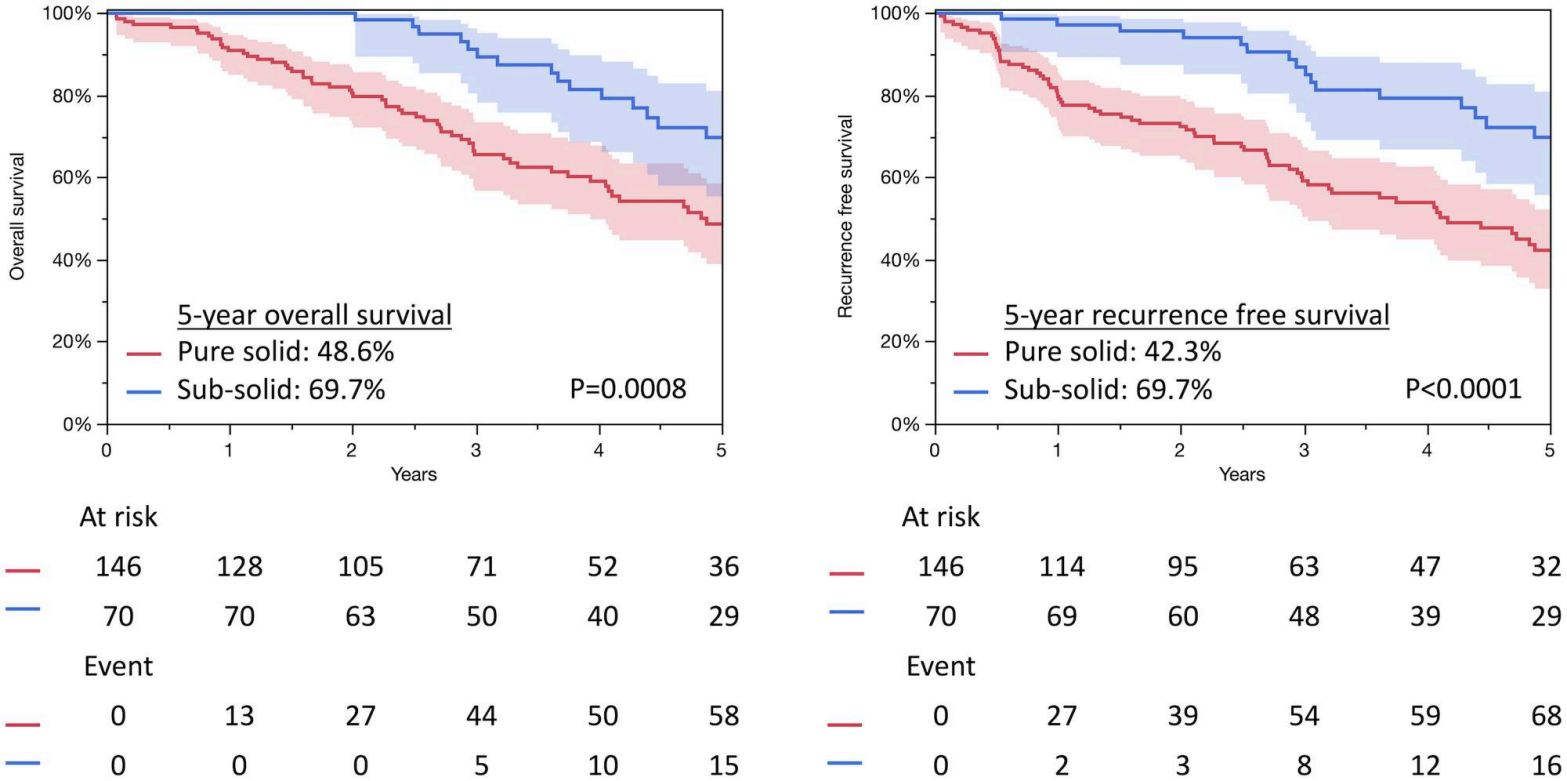
Overall Survival (A) and Recurrence Free Survival (B) Between Pure Solid and Subsolid Tumors. The coloured area indicates the 95% confidence interval

**Figure 2.**
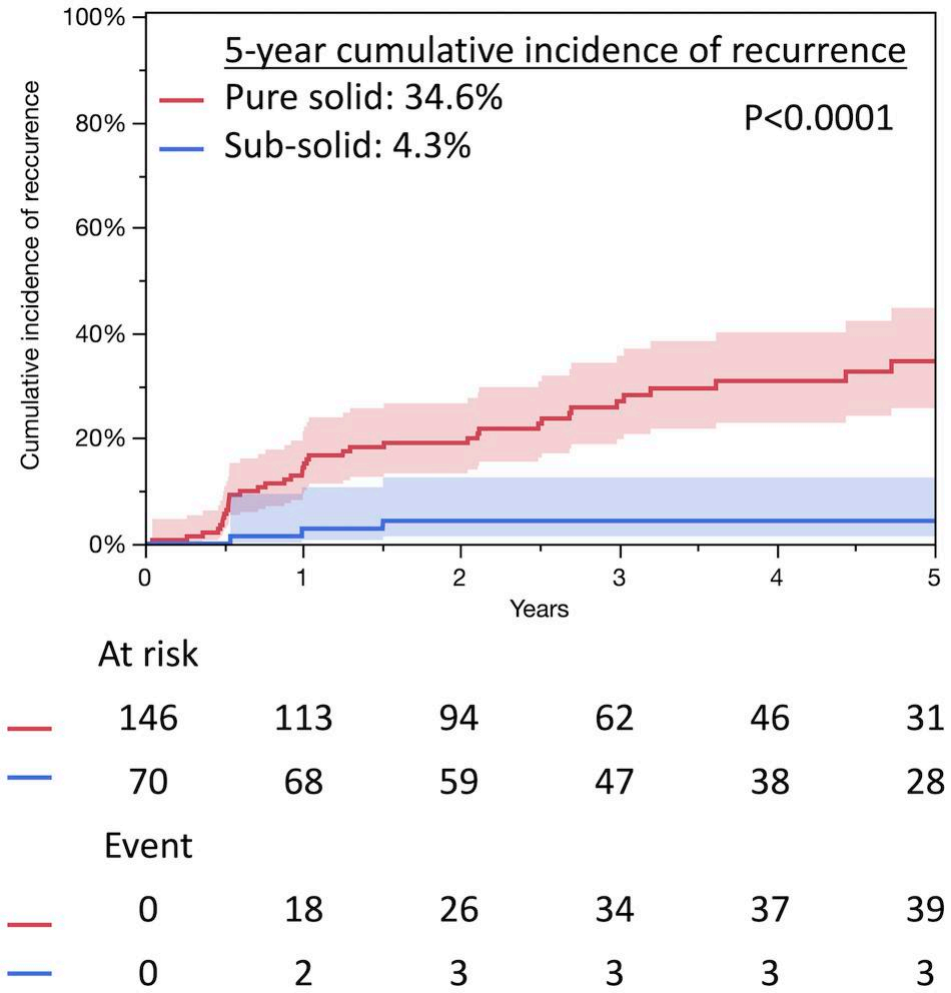
Cumulative Incidence of Recurrence Between Pure Solid and Subsolid Tumors. The coloured area indicates the 95% confidence interval

**Figure 3.**
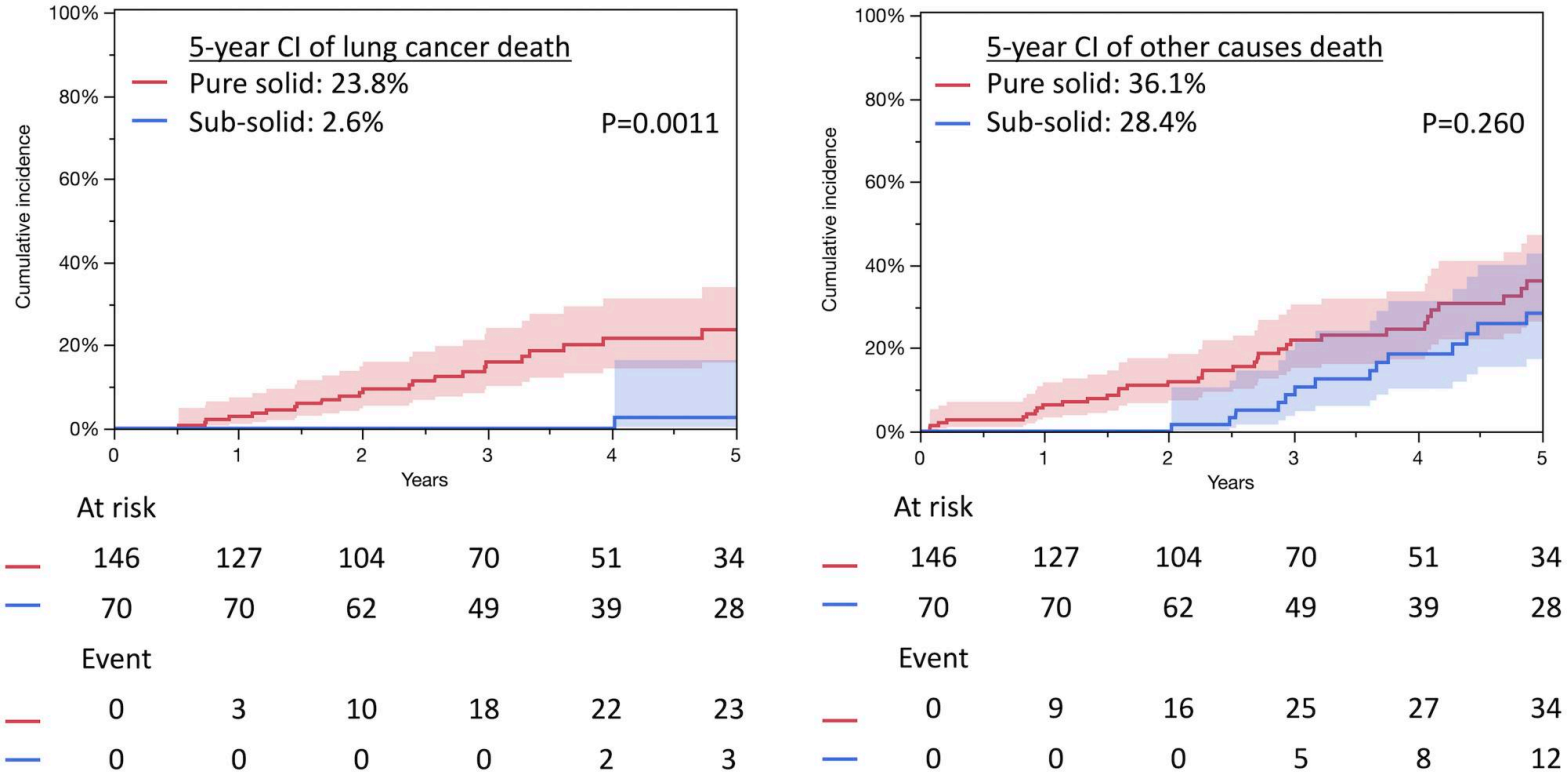
Cumulative Incidence of Lung Cancer Death (A) and Other Causes of Death (B) Between Pure Solid and Subsolid Tumors. The coloured area indicates the 95% confidence interval. CI, Cumulative Incidence

The median follow-up duration after surgery was 4.0 years (interquartile range (IQR), 2.5-6.6 years). In patients with pure solid tumours, the median follow-up duration was 3.5 years (IQR, 2.1-6.1) and the 5-year follow-up rate was 41%. In those with subsolid tumours, the median follow-up duration was 4.6 years (IQR, 2.5-7.2) and the 5-year follow-up rate was 56%. Survival analysis demonstrated that patients with subsolid tumours had significantly better prognoses compared to those with pure solid tumours (5-year OS rates [95% confidence interval]: 69.7% [55.3-81.0] vs 48.6% [38.9-58.5], *P* = .0008; 5-year RFS rates: 69.7 [55.7-80.9] vs 42.3 [32.9-52.1]%, *P* < .0001, respectively) (**Figure 1**). The 5-year cumulative incidence (CI) of recurrence was significantly lower in patients with subsolid tumours than in those with pure solid tumours (4.3% [1.4-12.5] vs 34.6% [25.7-44.7], *P* < .0001, respectively) (**Figure 2**). Recurrence patterns between the 2 groups are shown in **Table 2**. The 5-year CI of lung cancer deaths was significantly lower in patients with subsolid tumours than in those with pure solid tumours (2.6% [0.4-16.5] vs 23.6% [15.8-34.0], *P* = .0011, respectively); however, the 5-year CI of other causes of death did not differ significantly between the groups (28.4% [17.4-42.8] vs 36.1% [26.4-47.3], *P* = .260, respectively) (**Figure 3**). Notably, 2 years post-surgery, an increase in other causes of mortality was observed in patients with subsolid tumours. Respiratory failure and secondary malignancies, including secondary primary lung cancer, were the most common causes of non-lung cancer-related deaths in both subsolid and pure solid tumours (**Table 3**). Multivariable Cox regression analysis was performed using the entire cohort (**Table 4**). The presence of GGO was not significantly associated with improved OS. Additionally, separate multivariable analysis was then conducted for patients with subsolid tumours to identify prognostic factors associated with OS in this subgroup (**Table S1**). Male sex, higher maximum standard uptake, and lower vital capacity were associated with worse OS in patients with subsolid tumours (*P* = .005, *P* = .010, *P* = .001, respectively).

**Table 2.** Recurrence Pattern

Recurrence pattern	Pure solid	Subsolid
	*n* = 146	*n* = 70
Recurrence	41 (28%)	4 (6%)
Locoregional	20	2
Distance	13	2
Both	8	0

**Table 3.** Details of Other Causes of Death

	Pure solid	Subsolid
	*n* = 146	*n* = 70
Other causes of death	28 (19%)	13 (19%)
Respiratory failure	13	4
Acute exacerbation of interstitial pneumonia	8	2
Pneumonia	5	2
Secondary lung cancer	1	2
Other malignant disease	7	4
Heart failure	2	–
Cerebral infarction	1	1
Peritonitis	1	–
Trauma	1	–
Unknown	2	2

**Table 4.** Univariable and Multivariable Cox Analysis for Overall Survival in Entire Cohort

	Univariable		Multivariable	
	HR (95% CI)	*P*-value	HR (95% CI)	*P*-value
Age^a^	1.05 (1.02-1.08)	0.0030	–	–
Sex (male/female)	3.11 (1.61-6.03)	0.0008	3.91 (1.86-8.24)	0.0003
Solid tumour size^a^	1.03 (0.99-1.06)	0.0924		
SUV max^a^	1.10 (1.06-1.15)	<0.0001	1.10 (1.06-1.16)	<0.0001
Sub-solid tumour	Ref		–	–
Pure solid tumour	2.38 (1.41-4.02)	0.0012	–	–
Forced expiratory volume in 1 s^a^	0.99 (0.99-1.00)	0.057	–	–
Vital capacity^a^	0.99 (0.99-1.00)	0.074	0.99 (0.99-1.00)	0.0002
Inconsistent with UIP pattern	Ref		Ref	
Possible UIP pattern	1.34 (0.77-2.33)	0.296	1.15 (0.64-2.05)	0.632
UIP pattern	3.02 (1.73-5.27)	<0.0001	1.78 (1.04-3.71)	0.017
Wedge resection	Ref		Ref	
Segmentectomy	0.79 (0.46-1.36)	0.400	1.54 (0.84-2.84)	0.0001
Lobectomy	0.31 (0.19-0.52)	<0.0001	0.43 (0.25-0.74)	0.0024

Abbreviations: CI, confidence interval; HR, Hazard ratio; SUVmax, maximum standard uptake value; UIP, unusual interstitial pneumonia.

a Continuous value.

### Prognosis between patients with and without ILA, according to tumour type

Overall survival was significantly poorer in patients with ILA than in those without ILA, both in the pure solid and subsolid tumour groups (**Figure S2**). Among patients with pure solid tumour, the cumulative incidence of recurrence was significantly higher in the ILA group compared to the non-ILA group among patients with pure solid tumours. However, in subsolid tumours, whereas it was similar between the 2 groups for subsolid tumours (**Figure S3**).

## DISCUSSION

Among the patients with clinical stage IA NSCLC and ILA, 32% had subsolid tumours. The OS was significantly better in patients with subsolid tumours than in those with pure solid tumours; however, this difference was not significant in multivariable analysis. The cause of death differed between the 2 groups. Lung cancer-related deaths due to subsolid tumours were rare, with most deaths occurring after 2 years due to other causes. In contrast, pure solid tumours showed both lung cancer-related and other causes of mortality at a consistent rate. These findings suggest that while subsolid tumours remain potentially curative, the management of comorbidities leading to other causes of mortality is crucial for long-term follow-up.

The presence of a GGO component is an indicator of favourable prognosis in NSCLC.^9^^,^^10^^,^^14^^,^^15^ The JCOG0201 supplemental analysis^10^ demonstrated that the 5-year OS and RFS of subsolid tumours were significantly better than those of pure solid tumours because of their lower malignancy (90.1% vs 84.5%, *P* < .0001 and 93.3% vs 68.6%, *P* < .0001, respectively) in clinical stage IA NSCLC. However, patients with ILA were excluded from this study. In contrast, our study specifically investigated clinical stage IA NSCLC in patients with ILA and found that subsolid tumours exhibited less aggressive biological behaviour than pure solid tumours. This was evidenced by a lower SUVmax, higher proportion of lepidic-predominant adenocarcinoma, and lower positive rates of pleural and lymphvascular invasion, resulting in the lower recurrence rates observed in subsolid tumours. This has led to a lower cumulative incidence of lung cancer-related deaths.

Interestingly, despite these favourable tumour characteristics and reduced recurrence, multivariable analysis demonstrated that the presence of GGO was not associated with improved OS in patients with ILA. This may be attributed to the increased incidence of late non-cancer-related deaths occurring more than 2 years postoperatively in subsolid tumours. Indeed, the 5-year OS of 69.7% in patients with ILA with subsolid tumours was notably lower than that of 94.2% in those without ILA, consistent with the previously reported rates of 90.1%-97%^10^^,^^16^ in those without ILA with subsolid tumours. Although the exact reason for the delayed increase in mortality due to other causes remains unclear, several factors may have contributed to it. Compared to pure solid tumours, subsolid tumours were associated with a lower prevalence of radiological UIP patterns and a higher predicted diffusing capacity for carbon monoxide. This suggests that the subsolid tumour group included patients with less severe interstitial pneumonia, allowing for a stable postoperative period. However, progressive interstitial diseases may lead to an increase in non-cancer-related deaths over time. Among other causes of death, respiratory failure and secondary malignancies, including secondary primary lung cancer, are frequent in both subsolid and pure solid tumours. Given these findings, strategies to suppress interstitial pneumonia progression and maintain pulmonary function are crucial, as a decline in pulmonary function is associated with an increased risk of death.^17^^,^^18^

Sublobar resection may play a key role in preserving pulmonary function,^3^^,^^19^ which is essential for reducing the risk of postoperative acute exacerbation compared to lobectomy,^8^ preventing respiratory failure, and improving the outcomes of secondary malignancy. Additionally, sublobar resection is known to reduce the risk of postoperative acute exacerbation compared to lobectomy. Evidence from patients without ILA further supports this approach. The JCOG0802/WJOG4607L trial^20^ revealed that segmentectomy, compared to lobectomy, enabled more intensive treatment for postoperative recurrences or second primary lung cancers, leading to a lower rate of other causes of death in the segmentectomy group. Moreover, in patients with ILA, sublobar resection better preserves pulmonary function more than lobectomy.^21^ Taken together, these findings suggest that subsolid tumours with low malignant potential in patients with ILA could be optimal candidates for sublobar resection. However, the efficacy of sublobar resection in patients with ILA remains uncertain, as previous clinical trials, including JCOG0802/WJOG4607L,^20^ JCOG1211,^22^ and CALGB140503,^23^ have excluded this population. A definitive conclusion awaits the results of JCOG1708,^24^ a randomized trial of sublobar resection vs lobectomy in patients with clinical stage I NSCLC and ILA.

This study had a few limitations. First, it was a retrospective study, and reasons for the decision regarding the surgical procedure was unknown, including potential selection bias. In addition, surgical quality metrics were not available in our database, which might also have affected prognosis. Second, the present study used the 2011 classifications for CT; however, updated classification based on 2018 guidelines^25^ may enable more precise subgroup analyses. As our primary focus was the prognostic impact of GGO in patients with ILA, we believe the influence of classification is limited in this study. Third, the relatively low 5-year follow-up rate and wide confidence intervals in multivariable analyses due to limited sample size could affect outcome accuracy. Another limitation is that we did not perform propensity score matching; therefore, residual confounding due to unmeasured factors may remain. Additionally, linearized event rates were not calculated, but we provided cumulative incidence curves and number-at-risk tables instead. Fourth, detailed data on lymph node dissection, including the number and station of lymph nodes dissected were not available in our database. Fifth, our database lacks detailed information on antifibrotic drugs use, which could influence pulmonary function or outcomes.^26–28^ Interstitial pneumonia itself is an independent risk factor for postoperative pulmonary function decline,^29^ and insufficient control of disease progression may adversely affect long-term prognosis. Finally, because GGO tumours are more frequently observed in East Asian populations, the generalizability of our findings to Western populations may be limited. These limitations should be considered when interpreting the results.

In conclusion, in patients with clinical stage IA NSCLC and ILA, subsolid tumours exhibit less aggressive biological behaviour and have a lower cumulative incidence of lung cancer-related death than pure solid tumours. However, other causes of death, particularly respiratory failure and secondary malignancies 2 years post-surgery, lead to poorer OS. Management of comorbidities may improve long-term prognosis. Therefore, a prospective study is required to establish definitive conclusions.

## Supplementary Material

ivaf260_Supplementary_Data

## Data Availability

The data underlying this study are available in this article and its online supplementary material.
