# Supplementary material for: Prognostic Impact of Ground-Glass Opacity in Clinical Stage IA Non-Small Cell Lung Cancer With Interstitial Lung Abnormalities
Source: Interdiscip Cardiovasc Thorac Surg. 2025 Oct 31;40(11):ivaf260. doi: 10.1093/icvts/ivaf260 (PMC12629231; doi:10.1093/icvts/ivaf260)
Supplement: ivaf260_Supplementary_Data [file ivaf260_supplementary_data.zip › Supplementary Table1 20250711.docx]

Supplementary Table1. Univariable and multivariable Cox analysis for overall survival in pure solid and subsolid tumors.

|  | Pure solid | | | | Subsolid | | | |
| --- | --- | --- | --- | --- | --- | --- | --- | --- |
|  | Univariable | | Multivariable | | Univariable | | Multivariable | |
|  | HR (95% CI) | P. value | HR (95% CI) | P. value | HR (95% CI) | P. value | HR (95% CI) | P. value |
| Age* | 1.04 (1.01–1.08) | .027 | – | – | 1.06 (0.99–1.14) | .077 | – | – |
| Sex (male/ female) | 3.05 (1.22–7.60) | .017 | 4.14 (1.51–11.13) | .005 | 2.16 (0.77–6.08) | .143 | 7.19 (1.83–28.26) | .005 |
| Solid tumor size* | 0.98 (0.94–1.03) | .465 | – | – | 1.06 (0.99–1.14) | .075 | – | – |
| SUV max* | 1.08 (1.03–1.14) | .002 | 1.09 (1.72–14.31) | .005 | 1.14 (1.02–1.25) | .008 | 1.19 (1.05–1.34) | .010 |
| Forced expiratory volume in 1 second* | 0.99 (0.99–1.00) | .129 | – | – | 0.99 (0.99–1.00) | .145 | – | – |
| Vital capacity* | 0.99 (0.99–1.00) | .102 | 0.99 (0.99–0.99) | .001 | 0.99 (0.99–1.00) | .132 | 0.99 (0.99–0.99) | .001 |
| Inconsistent with UIP pattern | Ref |  | Ref |  | Ref |  | – | – |
| Possible UIP pattern | 1.48 (0.74–2.92) | .267 | 1.16 (0.57–2.38) | .675 | 1.07 (0.39–2.88) | .892 | – | – |
| UIP pattern | 3.02 (1.55–5.87) | .001 | 2.37 (1.17–4.78) | .016 | 1.27 (0.27–5.87) | .761 | – | – |
| Wedge resection | Ref |  | Ref |  | Ref |  | – | – |
| Segmentectomy | 1.25 (0.64–2.44) | .506 | 2.54 (1.21–5.34) | .014 | 0.76 (0.25–2.35) | .640 | – | – |
| Lobectomy | 0.38 (0.21–0.66) | 0.001 | 0.48 (0.27–0.88) | 0.018 | 0.26 (0.07–0.92) | 0.036 | – | – |
| CI, confidence interval; HR, Hazard ratio; SUVmax, maximum standard uptake value; UIP, unusual interstitial pneumonia. *, Continuous value | | | | | | | | |
