# Supplementary figures and images for: Prognostic Impact of Ground-Glass Opacity in Clinical Stage IA Non-Small Cell Lung Cancer With Interstitial Lung Abnormalities
Source: Interdiscip Cardiovasc Thorac Surg. 2025 Oct 31;40(11):ivaf260. doi: 10.1093/icvts/ivaf260 (PMC12629231; doi:10.1093/icvts/ivaf260)

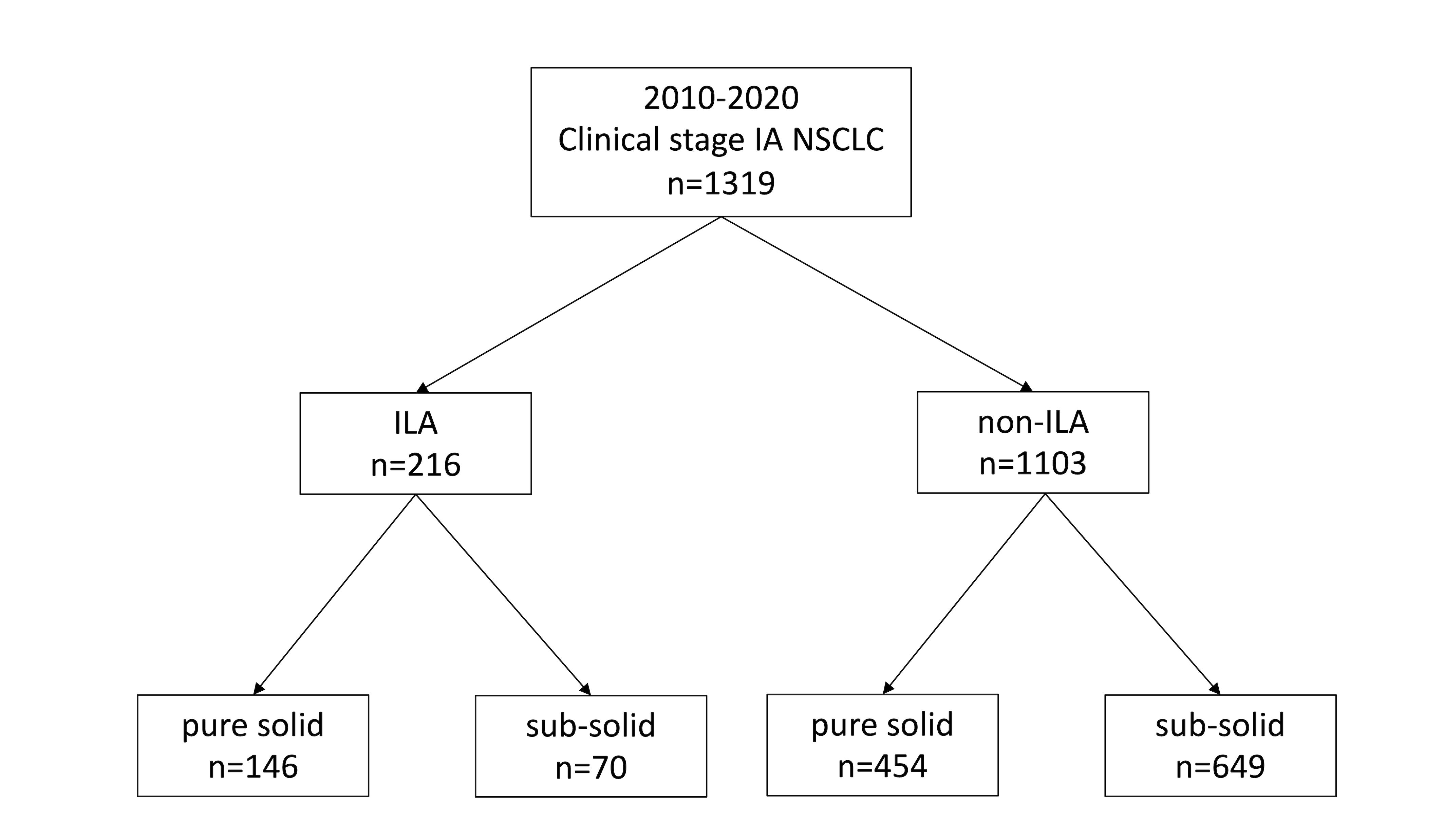

Supplement: ivaf260_Supplementary_Data [file ivaf260_supplementary_data.zip › FigureS1 Study flow.tiff]

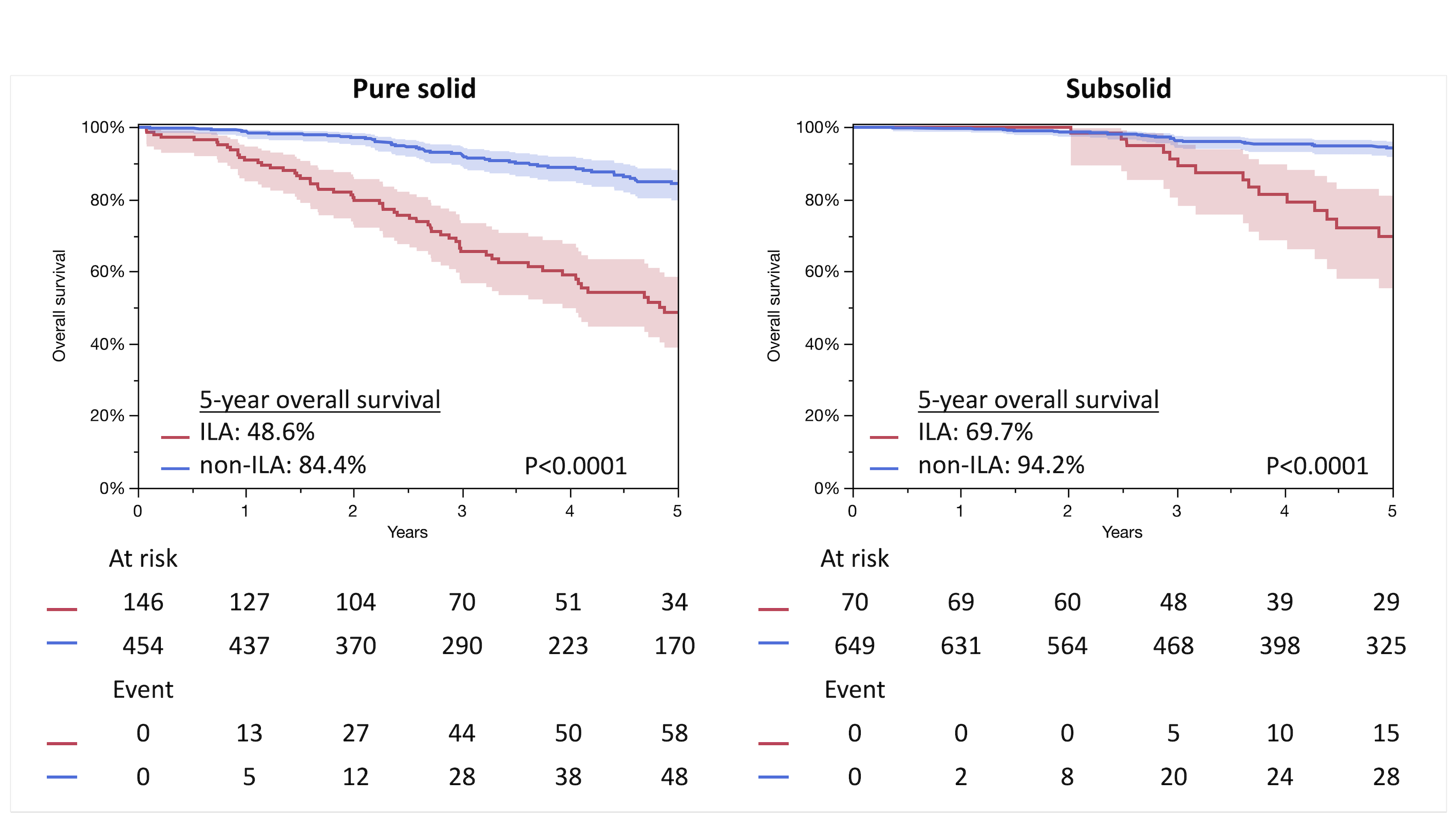

Supplement: ivaf260_Supplementary_Data [file ivaf260_supplementary_data.zip › FigureS2 Overall survival between ILA and non-ILA .tiff]

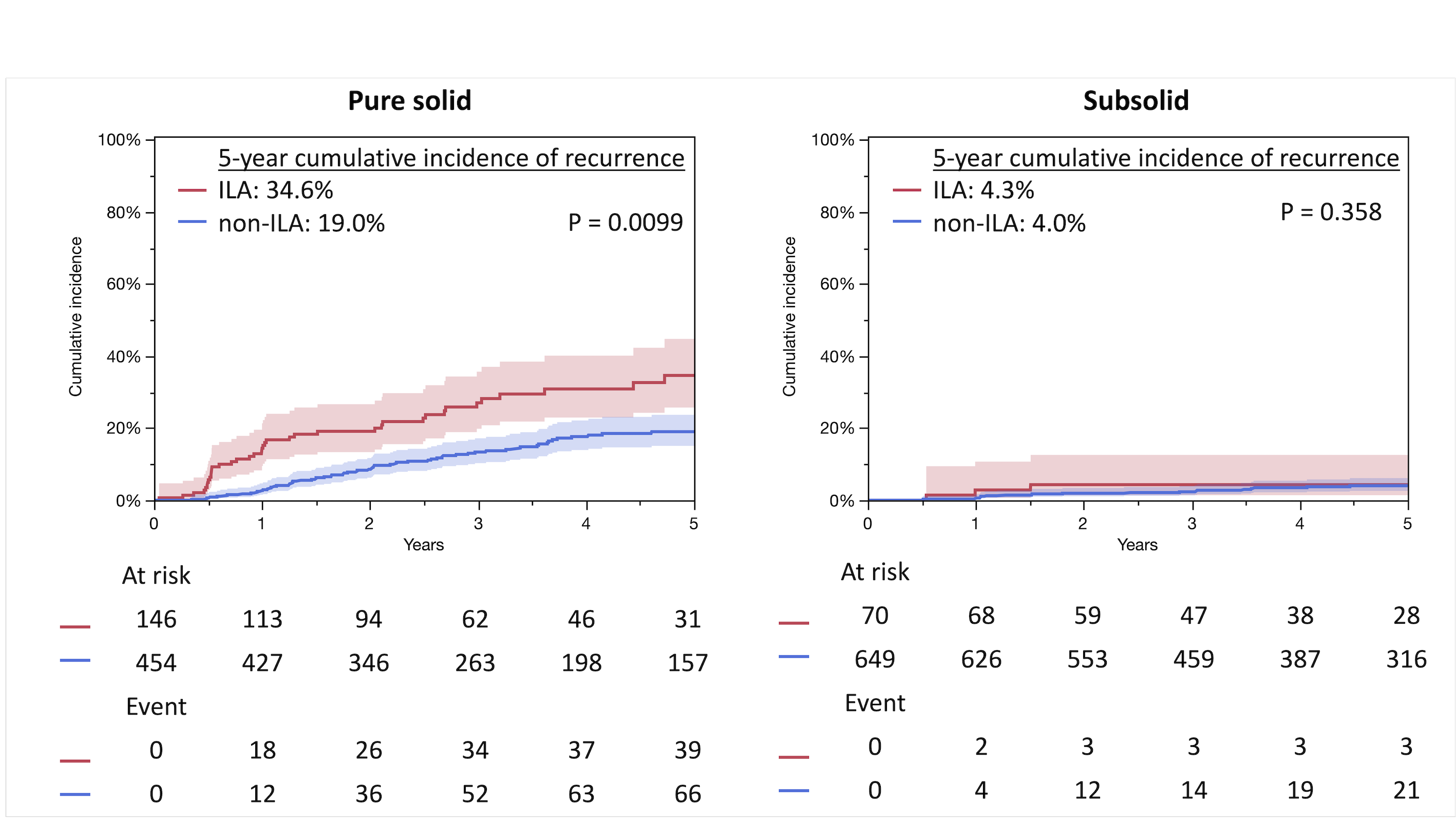

Supplement: ivaf260_Supplementary_Data [file ivaf260_supplementary_data.zip › FigureS3 CI of recurrence between ILA and non-ILA.tiff]
